# High-Quality *SiO*_2_/O-Terminated Diamond Interface: Band-Gap, Band-Offset and Interfacial Chemistry

**DOI:** 10.3390/nano12234125

**Published:** 2022-11-22

**Authors:** Jesús Cañas, Daniel F. Reyes, Alter Zakhtser, Christian Dussarrat, Takashi Teramoto, Marina Gutiérrez, Etienne Gheeraert

**Affiliations:** 1 Dpto. Ciencia de los Materiales, Universidad de Cadiz, 11510 Puerto Real, Spain; 2 Université Grenoble Alpes, CNRS, Grenoble INP, Institut Neel, 38000 Grenoble, France; 3 Instituto de Ciencia de Materiales de Sevilla (CSIC-Univ. Sevilla), Avda. Americo Vespucio 49, 41092 Sevilla, Spain; 4 Université Grenoble Alpes, CNRS, LTM, 38000 Grenoble, France; 5 Air Liquide Laboratories, Yokosuka 239-0847, Japan; 6 Japanese-French Laboratory for Semiconductor Physics and Technology J-F AST, CNRS, Université Grenoble Alpes, Grenoble INP, University of Tsukuba, Ibaraki 305-8577, Japan; 7 University of Tsukuba, Tsukuba 305-8573, Japan

**Keywords:** silicon oxide, diamond, interface, band-gap, band-offset, EELS, XPS

## Abstract

Silicon oxide atomic layer deposition synthesis development over the last few years has open the route to its use as a dielectric within diamond electronics. Its great band-gap makes it a promising material for the fabrication of diamond–metal–oxide field effects transistor gates. Having a sufficiently high barrier both for holes and electrons is mandatory to work in accumulation and inversion regimes without leakage currents, and no other oxide can fulfil this requisite due to the wide diamond band-gap. In this work, the heterojunction of atomic-layer-deposited silicon oxide and (100)-oriented p-type oxygen-terminated diamond is studied using scanning transmission electron microscopy in its energy loss spectroscopy mode and X-ray photoelectron spectroscopy. The amorphous phase of silicon oxide was successfully synthesized with a homogeneous band-gap of 9.4 eV. The interface between the oxide and diamond consisted mainly of single- and double-carbon-oxygen bonds with a low density of interface states and a straddling band setting with a 2.0 eV valence band-offset and 1.9 eV conduction band-offset.

## 1. Introduction

Ultra-wide band-gap semiconductors provide the material basis for building more efficient electronic devices thanks to their superior properties. Among them, diamond stands out with its outstanding breakdown field (up to 10 MV/cm [1]) and its high electron and hole mobilities, which ensure excellent performances [2]. Importantly, it also possesses the best thermal conductivity and an enormous radiation hardness. These properties make diamond devices interesting for high-temperature, high-power, and harsh environments applications [3,4,5].

Concerning diamond transistors, remarkable progress has been shown in the last two decades as a result of intensive research. The majority of the successful diamond transistors are built thanks to the 2D hole gas that is present on the hydrogen-terminated diamond surface. These H-terminated Field effect transistors (FETs) show good on-state characteristics as this approach solves the low carrier density at room temperature challenge present in diamond caused by the deep dopant levels available [6,7,8,9]. However, thermal and time stability remain the main challenges to overcome, even if different solutions have already been proposed [10,11]. Alternatively, transistors such as MESFETs [12,13,14], JFETs [15,16], and depletion MOSFETs [17,18,19,20] based on p-type diamond are also an interesting approach. High temperature stability and radiation hardness are their main advantage over H-terminated FETs [21]. Lastly, a low power demonstration of diamond inversion-based transistors [22] has been achieved, but a high power implementation is still lacking due to the still immature technology.

With the opposite behaviour to the hydrogen termination, the oxygen termination of diamond displays an insulating character. It is thermally stable up to 700 K and can be useful for passivation, as it depletes holes away from the surface [23,24,25]. Transistor gates based on this termination can be very useful to acquire a normally off behaviour in both surface [7] and bulk [14] conducting transistors. However, several oxides such as alumina, zirconia, or hafnia have been investigated on oxygen-terminated diamond, showing leakage currents, hysteresis, staggered band setting, and Fermi level pinning [26,27,28,29,30,31,32]. The most-promising oxide in this respect is silicon oxide, where the accumulation regime and low density of interface states were found in our previous work [33] and a straddling band setting was predicted by calculations [3]. Additionally, normally off operation has been achieved by MOSFETs based on this oxide [34,35].

In this work, atomic layer deposition (ALD) of $SiO_{2}$ is performed on p-type (100)-oriented O-terminated diamond in order to study the interfacial properties responsible for its low density of interface states [33]. A combined experimental study using scanning transmission electron microscopy in its energy loss spectroscopy mode (STEM-EELS) and X-ray photoelectron spectroscopy (XPS) to probe the band-gap of $SiO_{2}$ and the band alignment and interfacial chemistry between diamond and $SiO_{2}$ is presented.

STEM-EELS is a very strong tool to investigate the chemistry and band-gap at nanometre resolution, which is a necessity in the context of thin films. Nonetheless, the measurement of a material band-gap with valence EELS (VEELS) requires precise operation conditions and spectrum treatment to avoid an incorrect assessment. The single scattering distribution of electrons is directly related to the band-gap; however, multiple effects such as the Cherenkov effect or the zero loss peak are also present in the VEELS spectrum. To reduce the Cherenkov effect, which particularly affects the band-gap estimation, low acceleration voltages of 60 kV are needed for an accurate measurement of the band-gap [31,36,37,38]. XPS is an accessible, but key tool to study the interface bonds, band-gap, and band setting of heterostructures. It has already been used to quantify band-offsets and interfacial chemistry of different oxides and surface terminations of diamond heterostructures [11,32,39,40,41,42,43,44], but no contribution has yet reported the properties of $SiO_{2}$ on oxygen-terminated diamond.

## 2. Materials and Methods

**Figure 1:**
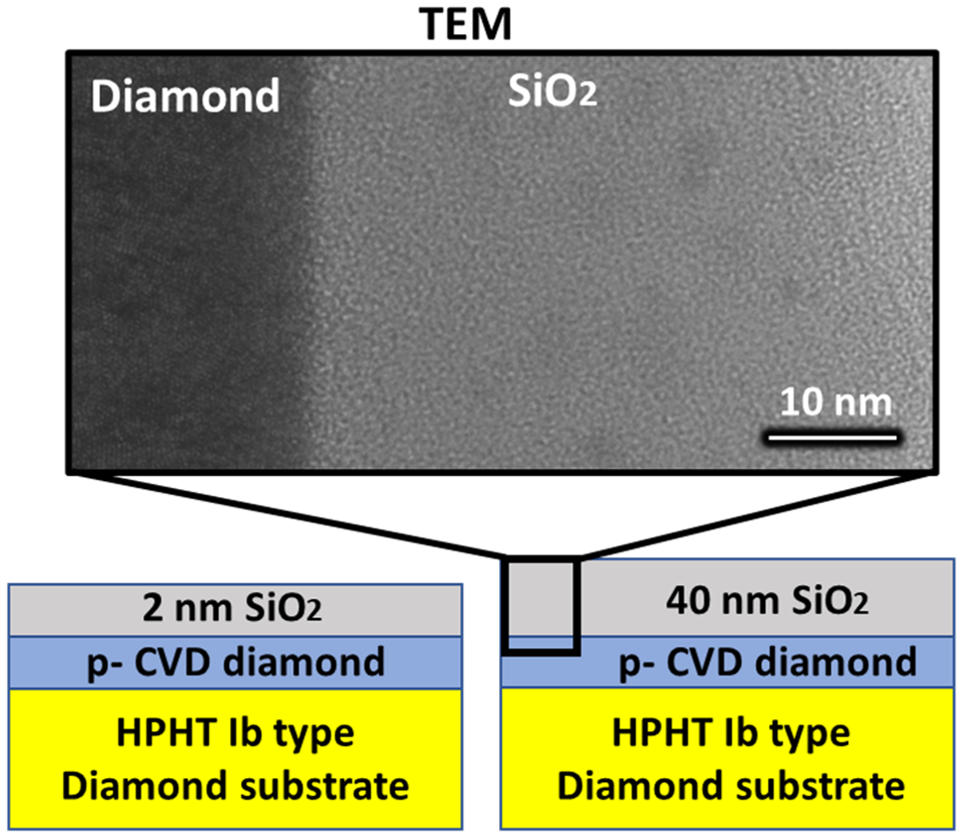
Scheme of the two analysed samples. The samples consist of silicon oxide layers of 2 nm and 40 nm deposited by ALD on boron-doped MPCVD diamond layers grown on High pressure high temperature (HPHT) Ib diamond substrates. A transmission electron microscopy image of the thicker 40 nm silicon oxide layer is also presented.

Two samples consisting of O-terminated CVD-grown (100)-oriented p-type diamond with $SiO_{2}$ layers grown by ALD were used in this work. First, a p-type diamond layer of about a 1 micron thickness was grown by MPCVD with a nominal boron concentration of about $10^{16}\;{\mathrm{cm}}^{-3}$ using $CH_{4}/H_{2}$ = 1%, $O_{2}/H_{2}$ = 0.25%, and B/C = 60 ppm at 900 ºC on a (100) Sumitomo Ib substrate. The pressure used was 33 Torr, and the microwave power was 240 W. After the epitaxy, an ozone plasma treatment was carried out with the objective of oxidizing the sample surface [24,45]. The treatment consisted of a 120 min ozone plasma at 500 mbar achieved by using a Xenon EXCIMER UV lamp at 172 nm. Subsequently, 2 nm and 40 nm of $SiO_{2}$ were grown by ALD on each of the samples. The ALD conditions were similar to those used in our previous contribution [33], where the close-to-ideal electric behaviour of this interface was shown. A full scheme of the samples together with a TEM image of the diamond–$SiO_{2}$ interface for the 40 nm sample are presented in Figure 1.

The resultant $SiO_{2}$ layer was studied by scanning transmission electron microscopy in electron energy loss mode. Concerning the lamella preparation, the sample was nano-machined with Ga+ in a Helios Nanolab 650 SEM-FIB. The specimen was characterized by using an FEI-TITAN THEMIS double-aberration-corrected electron microscope. An accelerating voltage of 60 kV was used in order to avoid the Cherenkov effect. A 0.12 eV FWHM zero loss peak (ZLP) was acquired by using a monochromator. A convergence semi-angle of 16.00 mrad and a collection semi-angle of 20.02 mrad were used to obtain the low-loss spectra at the diamond–$SiO_{2}$ interface. Concerning the XPS study, all the measurements were carried out using high-resolution monochromatic Al-Kα radiation (hv = 1486.7 eV). The spectra were recorded using a 0.1 eV step and a 60 eV pass energy after a mild Ar+ cleaning with low energy (0.5 kV) and an oblique angle (57°). The peak contributions were extracted by combining a Lorentzian and a Gaussian function (Voigt profile). The background was subtracted using the Tougaard background model function [46].

## 3. Results

**Figure 2:**
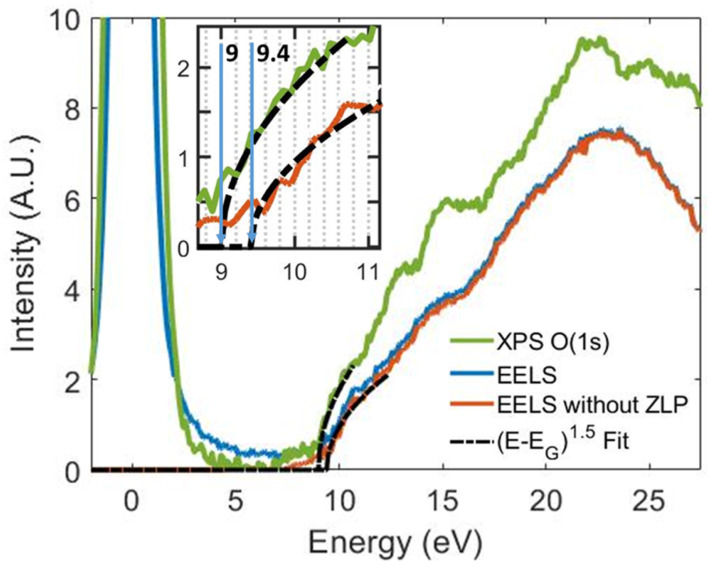
Scanning transmission electron microscopy—Valence energy loss spectroscopy (STEM-VEELS) spectrum measured on the 40 nm $SiO_{2}$ lamella represented as recorded (in blue) and with the zero loss peak deconvoluted (in red). The O(1s) X-ray photoelectron spectroscopy—photoelectron energy loss spectroscopy (XPS-PEELS) spectrum recorded on the 40 nm $SiO_{2}$ sample is represented in green with the O(1s) peak maximum as the energy origin. An ${\left(E-E_{gap}\right)}^{1/2}$ curve is used to fit the band-gap edge of both spectra and is represented with a black discontinuous curve. The fitting of the spectra edge is represented as well in the inset of the figure, yielding a 9.4 ± 0.2 eV band-gap for the VEELS spectrum and 9.0 ± 0.2 eV for the PEELS spectrum.

The VEELS spectrum recorded on the lamella of the 40 nm ALD-grown $SiO_{2}$ sample is presented in Figure 2 in blue. The VEELS spectrum is generally composed of a variety of phenomena such as the ZLP (0–2 eV), Cherenkov effect (4–6 eV), or plasmonic peaks, which can impede the assessment of the electronic transitions and band-gap determination. In this case, the ZLP contribution was deconvoluted using a logarithmic tail fit to the range 2 eV to 5 eV of the spectrum, leaving a plateau in the 5 to 8 eV region, as no contribution was expected at these energies. No Cherenkov-effect-related contribution was observed in the spectrum due to the low beam energy used (60 keV) and the relatively low dielectric constant of the $SiO_{2}$ layer. The ZPL-deconvolved spectrum is presented in red in Figure 2. The spectrum presents three clearly distinguishable inter-band transitions peaks at 10.7 eV, 14.8 eV, and 18.2 eV and a plasmon peak at 22.5 eV [47].

The single scattering distribution at the band-gap edge is proportional to a ${\left(E-E_{gap}\right)}^{n}$ curve. For the case of direct gap materials, this curve is proportional to the density of states, and so, an ${\left(E-E_{gap}\right)}^{1/2}$ curve was observed in the spectrum. Thus, by fitting an ${\left(E-E_{gap}\right)}^{n}$ curve, the value and the nature of the band-gap can be investigated. In Figure 2, the measurement of the $SiO_{2}$ band-gap can be observed for both the PEELS and VEELS spectra. The VEELS spectrum was accurately fit by a direct band-gap ${\left(E-E_{gap}\right)}^{1/2}$ curve, showing a band-gap value of 9.4 (±0.2) eV, which is in perfect agreement with reported values [47,48]. The small, but non-negligible contribution before the band-gap edge is associated with intra-gap transitions related to the surface of the lamella and, thus, is not representative of the thin film, but rather an artefact created by the preparation. The band-gap remained constant through the whole layer. The good agreement of the measurement within literature values together with the reproducibility among the layer ensures the good quality of the $SiO_{2}$ layer grown over the O-terminated (100)-oriented diamond.

In a similar fashion, the band-gap can be measured as well from the energy loss of the emitted photoelectrons when performing an XPS measurement. In Figure 2, the O(1s) photoelectron energy loss spectrum (PEELS) measured on the 40 nm $SiO_{2}$ sample is represented in green. The maximum of the O(1s) peak was set as the energy origin. The PEELS spectrum presents inter-band transitions peaks at 10.7 eV, 12.5 eV, 15.0 eV, and 17.9 eV and the plasmon peak at 22.2 eV. The similarities between the PEELS and VEELS spectra are remarkable with only the exception of the 12.5 eV peak not being distinguishable in the VEELS spectrum. The differences can be attributed to the higher surface sensitivity present in the PEELS measurement, as the collected scattered photoelectrons’ interaction region is roughly the 2–3 nm beneath the surface [49]. The PEELS spectrum was as well fit by a direct band-gap ${\left(E-E_{gap}\right)}^{1/2}$ curve, showing a band-gap value of 9.0 (±0.2) eV. Both techniques give compatible values within their error margins, although the VEELS measurement is considered more precise and reliable due to the reproducibility of the band-gap among spectra taken at different depths through the layer.

### 3.1. Band Adjustment and Interface Chemistry Determination

#### 3.1.1. Bulk $SiO_{2}$

**Figure 3:**
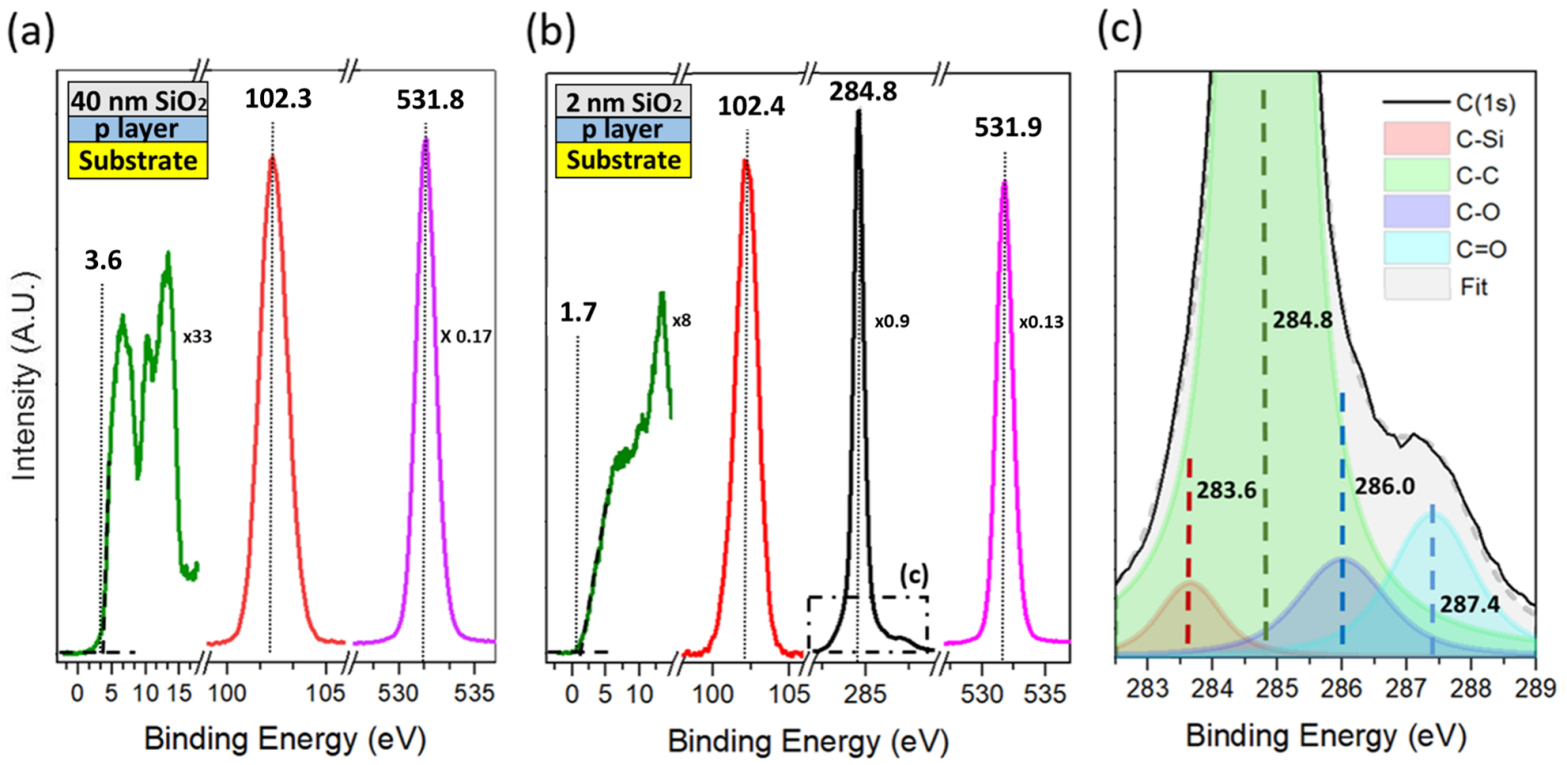
(**a**) XPS spectra of the VB, Si(2p), and O(1s) recorded on the 40 nm $SiO_{2}$ layer sample. (**b**) XPS spectra of the VB, Si(2p), C(1s), and O(1s) recorded on the 2 nm $SiO_{2}$ layer sample. The VB and peak heights are scaled for better comprehension of the reader in (**a**,**b**). The factors used to scale the VB and peak heights are displayed as well in (**a**,**b**) referenced to the Si(2p) height in each measurement. (**c**) XPS deconvoluted spectrum of the C(1s) peak recorded on the 2 nm $SiO_{2}$ layer sample.

The representative VB, Si(2p), and O(1s) XPS spectra from the measurement performed in the 40 nm sample are represented in Figure 3a. Since the inelastic mean free path of a photoelectron ejected from $SiO_{2}$ at ∼1 keV is about ∼3 nm [49], all the signal is unambiguously attributed to $SiO_{2}$. The lower energy range spectrum is attributed to the $SiO_{2}$ VB. The first three peaks at 6.4 eV, 10.3 eV, and 13 eV are characteristic of the amorphous phase of $SiO_{2}$. The valence band maximum (VBM) was determined by the widely used linear fit method [32], yielding a value of 3.6 eV. The peak centred at 102.3 eV is attributed to the Si (2p) spectrum. The whole Si(2p) area is attributed to Si bonded to oxygen (Si-O). Lastly, the O (1s) spectrum centred at 531.7 eV is shown. The summary of all the XPS peaks is presented in Table 1. Analogously, the whole area of this peak is attributed to oxygen bonded to silicon atoms (O-Si). The distance from Si(2p) to O(1s) is 429.4 eV, which is similar to reported values for the amorphous phase of $SiO_{2}$. By using the relative sensitivity factor (RSF), the relation between oxygen and silicon core levels provides a stoichiometry for the silicon oxide layer of 32.7% for silicon and 67.3 % for oxygen. This is very close to the 2:1 stoichiometry expected for the layer.

#### 3.1.2. Diamond–$SiO_{2}$ Interface

The representative VB, Si(2p), O(1s), and C(1s) XPS spectra from the measurement performed in the 2 nm sample are represented in Figure 3b. The lower-energy-range spectrum is attributed to the overlap of the $SiO_{2}$ and diamond VB. The peak at 14 eV is characteristic of diamond [32]. The VBM was determined again using the linear fit method [39], and the VBM was estimated as 1.7 eV. Thanks to the bigger contribution of diamond to the VB spectrum and the big valence band-offset between the two materials, the edge of the VB is attributed solely to diamond. The peak centred at 102.4 eV is attributed to the Si(2p) spectrum, while the peak centred at 531.9 eV to the O(1s) spectrum. Although an interfacial contribution is expected (whether it is C-O or Si-C), the whole Si(2p) and O(1s) areas are again attributed to Si-O bonding as there is no clear contribution of interface-related peaks in the spectra. An oxygen-rich stoichiometry of 28.8% of silicon and 71.2% oxygen was calculated with the relation of the peak areas by means of the RSF. The absence of clear interfacial peaks is explained by their proximity in energies to the main Si-O component in the Si(2p) and O(1s) spectra. The non-deconvoluted oxygen from the interface contributes positively to the extracted oxygen-rich silicon oxide layer stoichiometry as an artefact. Even so, the deviation from the 1:2 stoichiometry is most probably meaningful and related to the $SiO_{2}$ nucleation process.

Lastly, the C(1s) spectrum was found centred at 284.8 eV with smaller peaks at higher binding energies. These smaller peaks are attributed to interfacial bonding between diamond and $SiO_{2}$ and are shown in more detail in Figure 3c. A deconvolution of the C(1s) peak shows the presence of C-O (286 eV), C=O (287.4 eV), and C-Si (283.6 eV) bonding at the interface. The summary of all the XPS peaks, as well as the ratio of the interfacial peak areas are presented in Table 1. The relation between the areas shows a prominent carbon-oxygen bonding consisting of single- and double-bonding with a scarce silicon-carbon bond percentage in contrast with the work [11].

**Figure 4:**
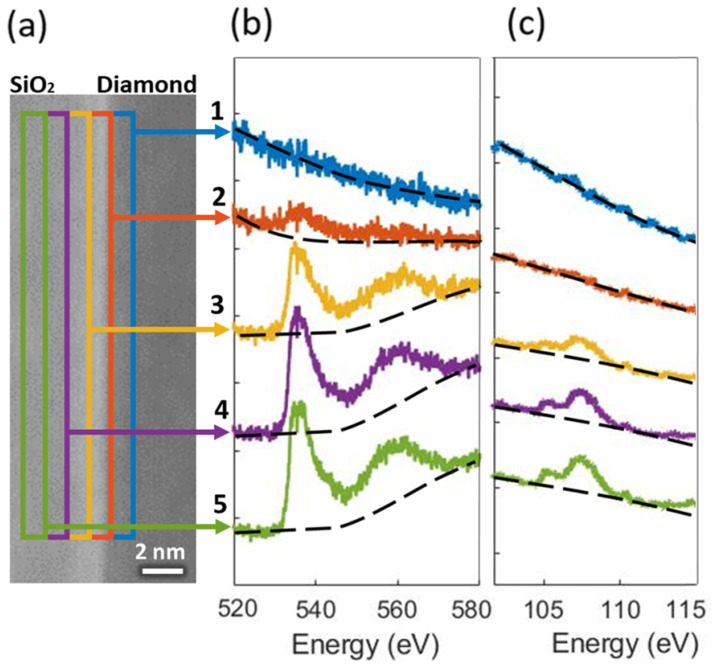
(**a**) ADF image of the $SiO_{2}$–diamond interface displaying the analysis regions for EELS. EELS spectrum of (**b**) O K-edge and (**c**) Si L-edge in the near-interfacial region.

The interface between diamond and $SiO_{2}$ was also studied by STEM-EELS on the lamella of the 40 nm ALD-grown $SiO_{2}$ sample. In Figure 4a, an annular dark field (ADF) image of the interface displaying the analysis regions for EELS is presented. The EELS spectra of the O K-edge and the Si L-edge are presented in Figure 4b,c respectively. The O K-edge and Si L-edge areas’ ratio is constant through the whole layer, indicating a constant stoichiometry. Even close to the interface, as in Spectrum 3, this ratio remains approximately constant. Spectrum 2, just at the interface, shows only features of oxygen, but no silicon, indicating C-O bonding, rather than C-Si bonding at the interface. Spectrum 1 is located at the diamond side, and both signals disappear as no oxygen or silicon was detected. These results support the conclusions extracted from the XPS analysis, pointing to a majority of C-O bonding at the interface, rather than C-Si bonds.

#### 3.1.3. Band Setting

The valence band-offset (VBO) between $SiO_{2}$ and diamond can be calculated thanks to the XPS spectra of their representative core peaks and their VBM using the following formula [39]:

$$VBO=\left(Si{\left(2p\right)}_{SiO_{2}}^{40nm}-VBM_{SiO_{2}}^{40nm}\right)-\left(Si{\left(2p\right)}_{SiO_{2}}^{2nm}-C{\left(1s\right)}^{2nm}\right)-\left(C{\left(1s\right)}_{diam}^{O-diam}-VBM_{diam}^{O-diam}\right)$$

where the first term is the distance between the Si(2p) core level and the VBM measured in the 40 nm-thick sample, the second term is the distance between the Si(2p) core level and the C(1s) core level measured in the 2 nm thin sample, and the third term is the distance between the C(1s) core level and the VBM measured in a O-terminated diamond sample. Applying the values from Table 1 and the most reliable C(1s)-VBM value reported in the literature of 282.8 eV [50], a value for the VBO of 1.7 eV was extracted. However, the great dispersion of values found in the literature for the C(1s)-VBM term encourages the estimation of the VBO using the following formula, as in our previous work [32]:

(1) $$VBO=\left(Si{\left(2p\right)}_{SiO_{2}}^{40nm}-VBM_{SiO_{2}}^{40nm}\right)-\left(Si{\left(2p\right)}_{SiO_{2}}^{2nm}-VBM_{diam}^{2nm}\right)$$

where the first term is the same as in the previous approach, but the second term is the distance between the Si(2p) core level and the (diamond) VBM directly measured in the 2 nm $SiO_{2}$ layer sample. This approach can be taken due to the big offset between diamond and silicon oxide, allowing the valence band maximum of diamond to be probed directly through the 2 nm of $SiO_{2}$.

**Figure 5:**
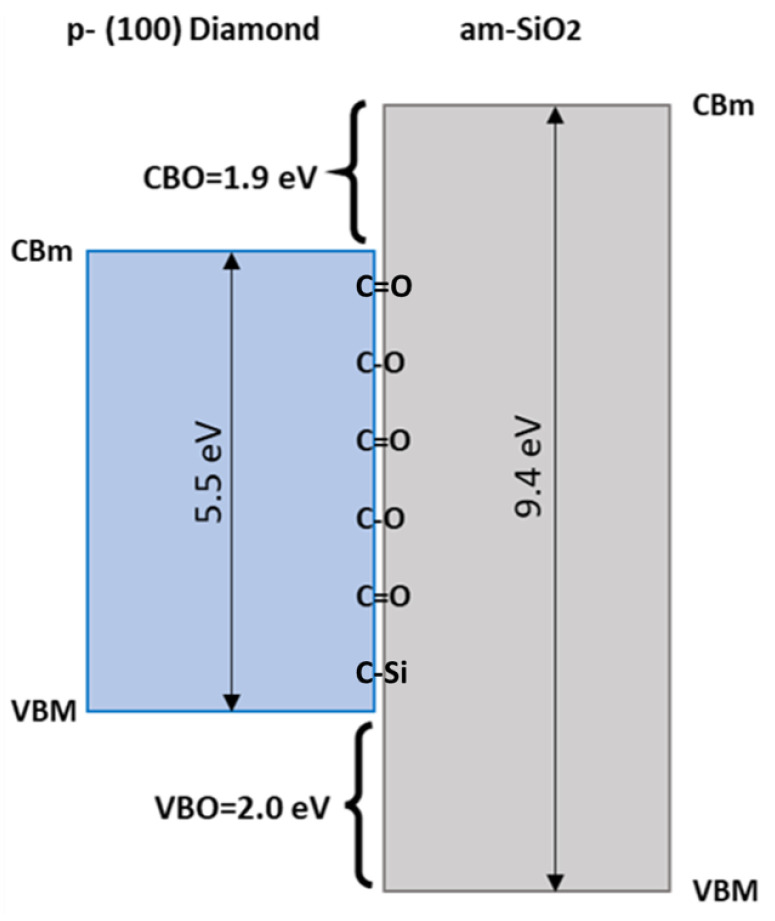
Scheme of the band alignment between $SiO_{2}$ and O-terminated (100)-oriented p-type diamond showing its interfacial chemistry.

Using the data provided in the Table 1, a value of 2.0 eV was deduced, in relatively good agreement with the previously deduced value, thus validating this latter approach. Finally, applying the value of the $SiO_{2}$ layer band-gap measured by VEELS, a conduction band-offset (CBO) of 1.9 eV was extracted. Therefore, a straddling band setting was deduced between diamond and $SiO_{2}$. A scheme of the band alignment and the interfacial chemistry is presented in Figure 5.

The theoretical VBO value reported between $SiO_{2}$ and O-terminated diamond deduced from their electron affinities is 2.7 eV [51], which is higher than the value extracted here. As the electron affinity approach does not take into account the formation of bonds at the interfaces and their associated charge redistribution, the difference between theoretical and experimental values can be explained as a result of the new bonding configuration and its resulting interface dipolar contribution to the band alignment [52]. Despite this, this measurement shows encouraging results as substantially big offsets are reported for the conduction and valence bands. Particularly noteworthy is the existence of a high barrier for electrons since no other oxide has been reported to have any, using similar experimental techniques to the one reported here, even though inversion regimes have been demonstrated on p-type diamond MOSFETs based on alumina [22]. These results open the route of the ALD $SiO_{2}$–O-diamond interface for fabricating the gate of diamond transistors, whether if it is for inversion-based MOSFETs, due to the measured straddling band setting with a large electron barrier, or normally off H-MOSFETs, due to the low interface states’ density [33] and the fabrication ease of this interface.

## 4. Conclusions

A detailed investigation of the properties of the ALD-grown $SiO_{2}$ and oxygen-terminated p-type (100)-oriented diamond heterojunction was presented in this work. The band-gap of the amorphous $SiO_{2}$ is reported to be constant among the layers on a nanometric level with a value of 9.4 eV. The oxide bonding with diamond mainly consists of double- and single-C-O bonds, and the heterojunction presents a straddling band setting with 2.0 eV VBO and 1.9 eV CBO. These results show the potential of $SiO_{2}$ for the successful fabrication of diamond MOSFET gates where barriers both for holes and electron are present in contrast with any oxide ever investigated with similar techniques.

## Figures and Tables

**Table 1:** Summary of the XPS peaks’ energy and full-width at half-maximum (FWHM) for the two studied samples (2 nm and 40 nm of $SiO_{2}$). The C(1s) deconvoluted interfacial components’ (measured on the 2 nm $SiO_{2}$ sample) energies and relative areas’ (RA) are also shown at the bottom of the table. The RA is calculated as the ratio of the area between one of the interfacial component over the sum of the areas of all of them.

**Spectrum**		**2 nm ** ${\mathrm{SiO}}_{2}$	**40 nm ** ${\mathrm{SiO}}_{2}$
VBM	Energy	1.7 eV	3.6 eV
Si(2p)	Energy	102.4 eV	102.3 eV
	FWHM	1.7 eV	1.6 eV
O(1s)	Energy	531.9 eV	531.8 eV
	FWHM	1.6 eV	1.5 eV
C(1s)	Energy	284.8 eV	-
	FWHM	0.7 eV	-
**C(1s) interfacial component**		**Energy**	**Relative area**
** $C{\left(1s\right)}_{C-Si}$ **		283.6 eV	15%
** $C{\left(1s\right)}_{C-O}$ **		286.0 eV	35%
** $C{\left(1s\right)}_{C=O}$ **		287.4 eV	50%

## Data Availability

The data that support the findings of this study are available from the corresponding author upon reasonable request.
